# Recent Advances in Crosslinked Nanogel for Multimodal Imaging and Cancer Therapy

**DOI:** 10.3390/polym12091902

**Published:** 2020-08-24

**Authors:** Wen Zhou, Guangzhao Yang, Xiaoyue Ni, Shanchao Diao, Chen Xie, Quli Fan

**Affiliations:** Key Laboratory for Organic Electronics and Information Displays, Institute of Advanced Materials (IAM), Jiangsu National Synergetic Innovation Center for Advanced Materials (SICAM), Nanjing University of Posts & Telecommunications, Nanjing 210023, China; wenzhou@nankai.edu.cn (W.Z.); 1019061525@njupt.edu.cn (G.Y.); 1019061402@njupt.edu.cn (X.N.); 1219064405@njupt.edu.cn (S.D.)

**Keywords:** crosslinked nanogel, cancer therapy, multimodal imaging, phototheranostics

## Abstract

Nanomaterials have been widely applied in the field of cancer imaging and therapy. However, conventional nanoparticles including micelles and liposomes may suffer the issue of dissociation in the circulation. In contrast, crosslinked nanogels the structures of which are covalently crosslinked have better physiological stability than micelles and liposomes, making them more suitable for cancer theranostics. In this review, we summarize recent advances in crosslinked nanogels for cancer imaging and therapy. The applications of nanogels in drug and gene delivery as well as development of novel cancer therapeutic methods are first introduced, followed by the introduction of applications in optical and multimodal imaging, and imaging-guided cancer therapy. The conclusion and future direction in this field are discussed at the end of this review.

## 1. Introduction

Cancer is one of the major threats to human lives all over the world [1]. Traditional cancer treatment process divides diagnosis and therapy into different procedures, which is time consuming and requires high cost [2,3]. In contrast, cancer theranostics which combines diagnosis and therapy into one system can overcome such disadvantages, and has shown great potential in the field of cancer treatment [4,5,6]. Recently, phototheranostics, which based on optical imaging, have been widely studied because of their unique advantages such as high safety and sensitivity, low cost and capability of multi-channel imaging [7,8,9,10,11]. Numerous phototheranostic systems based on variety of materials such as small molecular dyes [12,13], anti-cancer drugs [14,15] and biomacromolecules [16] have been developed. However, as theranostics require the combination of multiple functionalities including imaging and therapy, complicated synthetic procedures are usually inevitable [17,18]. Therefore, developing phototheranostic systems based on multifunctional and facile synthesized materials is in high demand.

Among numerous materials for phototheranostics, nanomaterials have shown great promise and been widely applied in the field of cancer imaging and therapy [19,20]. Compared with small molecular dyes or drugs, nanomaterials can be easily prepared via nano-based preparation methods such as nanoprecipitation and nanoemulsion [21,22,23]. Some inorganic nanoparticles or 2D nanomaterials have unique optical properties, which can be used as phototheranostics directly [24,25,26,27]. In addition, different functionalities can be integrated into nanomaterials by simply doping or linking different moieties [28,29]. Anti-cancer drugs can also be absorbed or covalently linked onto such nanomaterials, endowing them with the capability for chemotherapy [30,31]. For organic nanomaterials, hydrophobic anti-cancer drugs or photosensitizers as well as optical imaging contrast agents can be encapsulated into micelles or liposomes simultaneously, which is a conventional way to construct phototheranostic platforms [32,33,34,35]. Owing to their relatively good biocompatibility, organic nanoparticles have gained increasing attention in the field of phototheranostics.

Although organic nanoparticles have been widely applied in cancer imaging and therapy, some drawbacks of conventional nanoparticles should be overcome. As most organic nanoparticles are micelles and liposomes, they may dissociate when their concentration decrease lower than the critical micelle concentration [36]. Such a feature makes nanoparticles unstable in harsh conditions such as blood circulation, which further leads to the burst release of encapsulated drugs or contrast agents [37,38]. To overcome such drawbacks, crosslinked nanogels have been chosen in the development of phototheranostic systems. Compared with micelles or liposomes, a crosslinked structure can stabilize the nanogels, leading to the non-dissociable nanostructure [39,40,41,42]. Such structure makes nanogels keep intact in the circulation, thus resulting in improved biodistribution and better tumor accumulation [43,44].

Until now, the application of micelles and liposomes in the field of cancer phototheranostics have been very well summarized [45,46,47]. However, the synthesis and properties of crosslinked nanogels as well as their applications in such field have rarely been reviewed. In this review, we summarize recent advances of crosslinked nanogels for cancer theranostics. The preparation methods, especially nanogel crosslinking pathways, are first introduced. Then, the application of nanogels in cancer therapy including chemotherapy, gene therapy and enzyme dynamic therapy are summarized, followed by the summary of applications in cancer theranostics. Finally, a brief summary is given with the discussion of current status and future perspectives in this field.

## 2. Preparation of Crosslinked Nanogels

**Figure 1:**
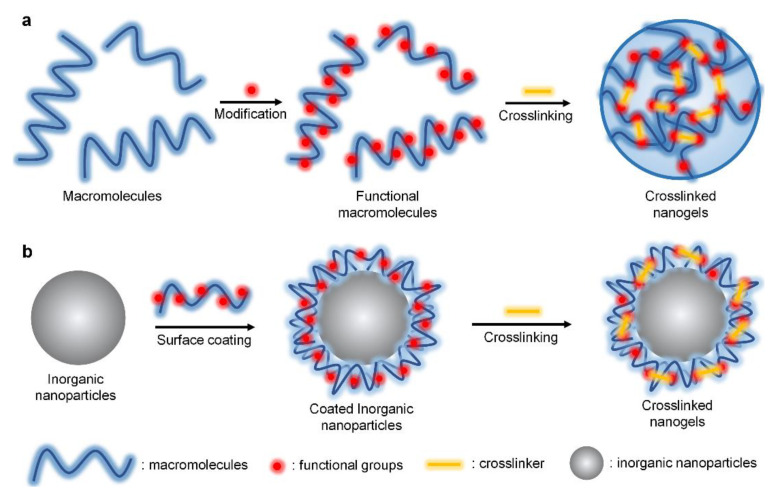
Schematic illustration for preparation of crosslinked nanogels. (**a**) Preparation of macromolecules-based crosslinked nanogels. (**b**) Preparation of inorganic nanoparticles-based crosslinked nanogels.

There are two major kinds of crosslinked nanogel, one is pure organic nanogels, another is inorganic nanoparticles coated with crosslinked organic shells (Figure 1). The pure organic nanogels can be prepared by crosslinking functional macromolecules, which are synthesized by modifying functional groups onto macromolecules (Figure 1a). The crosslinkers may have bi-functional or multi-functional groups, and the nanogels can be crosslinked via covalent bonds [48], supramolecular interactions [49], π–π stacking [50] and electrostatic interactions [51]. In addition, some crosslinkers contain environmentally responsive moieties such as disulfide bonds, endowing nanogels with responsiveness [52]. As pure inorganic nanoparticles may have poor biocompatibility and be unstable in physiological conditions, coating biocompatible organic macromolecules onto their surface is a rational choice to enhance the stability and biocompatibility (Figure 1b). Crosslinking the coated shell can further stabilize the nanoparticles and form inorganic nanoparticle-embedded crosslinked nanogels. Compared with pure inorganic nanoparticles, such nanogels have not only better stability, but also improved biodistribution [53].

**Scheme 1:**
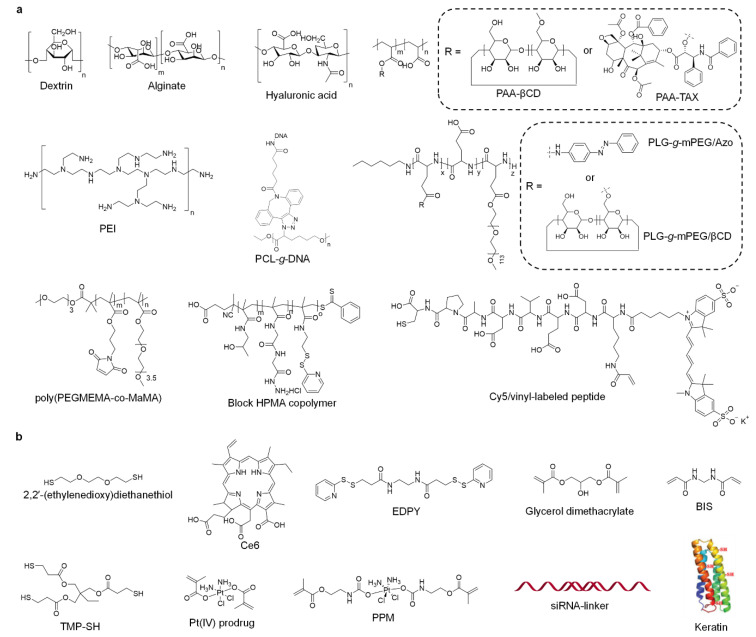
(**a**) Chemical structures of some macromolecules served as the backbone of nanogels. (**b**) Structures of some typical crosslinkers.

The preparation of nanogels introduced in this review is summarized in Table 1. The most commonly used materials for nanogel preparation are natural polysaccharides, such as dextrin, alginate and hyaluronic acid. These natural polymers not only have good biocompatibility, but also plenty of functional groups including hydroxyl and carboxyl groups which can be used as reactive sites for crosslinking. Other kinds of material are synthetic polymers with grafted functional groups. The structure of these polymers can be precisely controlled to achieve specific functionalities. For example, β-cyclodextrin (βCD) can be grafted onto the side chain of poly(acrylic acid) (PAA) or poly(L-glutamic acid) (PLG), and further form nanogels via supramolecular interactions. DNA can be grafted onto poly(ε-caprolactone) (PCL) to give PCL-*g*-DNA as carrier of siRNA. Other functional groups such as maleimide and a disulfide bond can also be modified onto polymers, which can be crosslinked by click and disulfide exchange reactions. Polymerization among vinyl groups is also a conventional method to prepare crosslinked nanogels. Vinyl-labeled peptide is synthesized which can be further crosslinked by a polymerization reaction (Scheme 1a).

For crosslinkers, they must have two or more functional groups to crosslink different polymer chains together. In addition, the reactions chosen for crosslinking should have relatively high reaction yield. The most widely used reactions are some click-type reactions such as thiol-ene reaction, and thiol containing compounds can be used as crosslinkers. These reactions have very high yield and can be carried out under mild conditions. Disulfide exchange reaction is a good choice to construct nanogels with glutathione (GSH) responsiveness, as such reaction may form disulfide bonds in nanogels. Some disulfide pyridine containing crosslinkers can be chosen for developing such a kind of nanogel. Using polymerization reaction to crosslink macromolecules is a facile approach to prepare crosslinked nanogels. Such nanogels can be synthesized by using crosslinkers with two vinyl groups. However, such a method makes it difficult to develop nanogels with environmental responsiveness. To develop nanogels with high drug loading content, prodrugs can be designed as crosslinkers so that the drug can be incorporated into nanogels. Similarly, therapeutic siRNA can also be used as crosslinkers (Scheme 1b).

## 3. Crosslinked Nanogels for Cancer Therapy

Nanomaterials have been widely used as carriers for drug and gene delivery in the field of cancer therapy [75]. As crosslinked nanogels have the advantages including good stability and environmental responsiveness, nanogels can be good candidates for nanocarriers [76]. The crosslinked structure may prevent the burst release of loaded drugs. In addition, an activatable drug- or gene-delivery system can be developed by nanogels with environmental responsiveness [77]. In this section, we summarize applications of nanogels for cancer therapy including chemotherapy, gene therapy and enzyme dynamic therapy. The properties of these nanogels introduced in this section are summarized in Table 2. The most commonly used anti-cancer drug was doxorubicin (DOX), and it can be loaded via electrostatic interaction with nanogels. Drugs can be released under specific responsiveness such as pH, glutathione (GSH) and esterase. However, such method sometimes led to low drug loading capacity. To improve the drug-loading capacity, drug-crosslinked nanogels were designed, and the drug loading capacity can reach as high as 60.8%. For gene therapy, therapeutic RNAs were commonly loaded by ionic interaction, and can be released in a tumor-associated microenvironment.

### 3.1. Chemotherapy

Chemotherapy is still the major therapeutic method in cancer therapy. In the past decades, nanomedicines have showed great promise in the field of cancer therapy owing to their unique advantages such as high tumor accumulation, high drug loading capacity and low toxicity [78]. Numerous studies have utilized crosslinked nanogels for the drug carriers and showed satisfactory therapeutic efficiency against tumor.

A simple but efficient nanogel was designed by Cui et al. for combination therapy [54]. Such nanogels utilized alginate (ALG) as backbone, and were crosslinked by a calcium ion through ionic interaction. Glycyrrhizin (GL) and DOX were then loaded into nanogel via hydrogen bond and ionic bond, respectively. DOX can be released under acidic condition, which matches the microenvironment of tumor tissue. In addition, the loaded GL can reduce the phagocytosis of macrophages for nanogel, thus increasing the tumor accumulation of nanogels. In vivo studies indicated that the combination therapy of GL and DOX induced by nanogels can enhance the therapeutic efficiency. Micelle crosslinking can improve the stability of micelles and form nanogels which can be used as drug carriers [79]. Malkoch et al. designed a core-crosslinked nanogel for loading and delivering DOX [55]. Such nanogels were composed of a hydrophobic dendritic block as core and poly(ethylene glycol) (PEG) as shell. The dendritic core was then crosslinked via a thiol-ene click reaction to form dendritic nanogels (DNGs). DOX can be loaded onto DNGs and the drug-loaded DNGs showed good anti-cancer efficiency against 3D spheroid tumor cell model.

#### 3.1.1. Environmentally Responsive Nanogels for Chemotherapy

**Figure 2:**
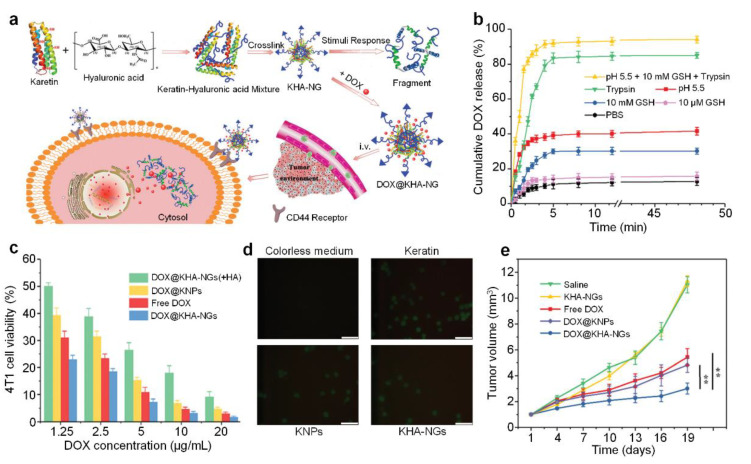
(**a**) Schematic illustration of preparation of KHA-NG and DOX@KHA-NG for cancer therapy. (**b**) DOX release profile of DOX@KHA-NGs under different conditions. (**c**) 4T1 Cell viability after being incubated with DOX, DOX@KNPs, DOX@KHA-NGs and DOX@KHA-NGs pre-treated with HA. (**d**) Intracellular NO concentration under different treatment. NO was detected by 3-amino,4-aminomethyl-2′,7′-difluorescein. Scale bars represent 100 μm. (**e**) Change of tumor volume of mice with post-treatment time under different treatments. ** *p* < 0.01. Adapted from [56]. Copyright© 2018 The Royal Society of Chemistry.

Compared with conventional nanogels-based drug-delivery systems (DDS), nanogels with environmental responsiveness may be a better choice for DDS development. The tumor microenvironment has features such as acidic pH, reactive oxygen species (ROS) and GSH overexpression [12]. Thus, development of nanogels which can response to these features is feasible method in DDS design. Li et al. designed a simple nanogels (KHA-NGs) composed of keratin and hyaluronic acid (HA) through a crosslinking method [56]. Keratin and HA were first mixed, and crosslinked with thiourea to form KHA-NGs. In the presence of trypsin or GSH, KHA-NGs can be degraded, which makes KHA-NGs a good stimuli-responsive drug carrier. DOX can be loaded into KHA-NGs to give DOX@KHA-NGs. In the tumor tissue, HA on the surface of nanogels can bind with the CD44 receptor, helping nanogels be internalized into cells, thus releasing drugs and kill cancer cells (Figure 2a). Transmission electron microscopy (TEM) images showed that DOX@KHA-NGs had a spherical morphology, while its hydrodynamic size was determined as 80 nm by dynamic light scattering (DLS). After loading DOX, the zeta potential of KHA-NGs increased dramatically, and the drug-loading capacity can reach 51.4%. The drug release profile was then studied. Under acidic condition with trypsin and GSH, DOX@KHA-NGs had the fastest DOX release, which was higher than any other single stimulus (Figure 2b). TEM images indicated that the nanogels became fragments under these stimuli. Cellular uptake of DOX@KHA-NGs was then studied by confocal fluorescence imaging. Strong fluorescence of DOX can be detected within cells treated with DOX@KHA-NGs, indicating that nanogels can be effectively internalized into cells. When pre-treating cells with free HA, the cell internalization was significantly inhibited. The in vitro anti-cancer effect was then studied, and DOX@KHA-NGs showed the highest anti-cancer efficacy to 4T1 cells compared with free DOX and DOX-loaded keratin nanoparticles (DOX@KNPs) (Figure 2c). Keratin was reported to stimulate nitric oxide (NO) release within cells. The NO concentration within nanogel-treated cells was then detected by a fluorescent indicator. The results indicated that keratin, KNPs and KHA-NGs can induce the generation of NO within 4T1 cells (Figure 2d). The nanogels were then be applied for in vivo cancer therapy. Semi-quantitative analysis showed that DOX had the highest accumulation into tumor site for DOX@KHA-NGs-injected mice. The change of tumor volume after treatment also indicated that DOX@KHA-NGs had best anti-cancer efficacy compared with other treatments (Figure 2e). Haemotoxylin and Eosin (H&E) staining demonstrated the safety of such treatment.

Disulfide bond is the most conventional moiety to develop GSH-responsive DDS [80]. However, the responsive rate of disulfide bond is relatively slow. To improve such disadvantage, Yu et al. designed a selenylsulfide bond (Se-S) grafted alginate, and crosslinked by magnetic nanoparticles to form nanogels [66]. Such nanogels can effectively load DOX and showed good stability under physiological conditions. The nanogels may be dissociate under acidic pH, and DOX can then be released in the presence of GSH. As Se–S had higher cleavage rate than the disulfide bond, such nanogels had relatively higher DOX release rate.

#### 3.1.2. Drug-Crosslinked Nanogels

**Figure 3:**
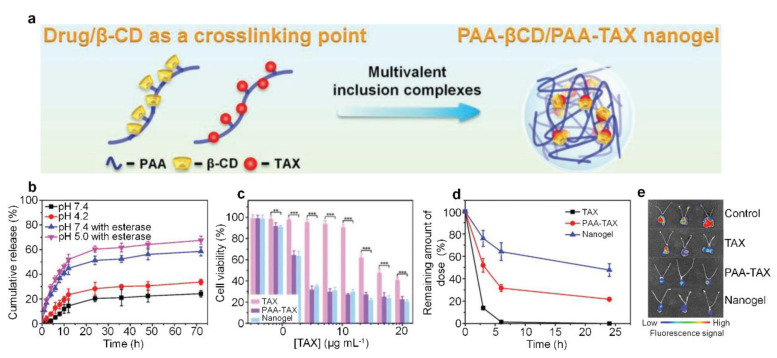
(**a**) Schematic illustration of formation of PAA-βCD/PAA-TAX nanogel. (**b**) In vitro drug release profile of PAA-βCD/PAA-TAX nanogel under different conditions. (**c**) In vitro cytotoxicity study of TAX, PAA-TAX and PAA-βCD/PAA-TAX nanogel against HeLa/TAX cells. ** *p* < 0.01; *** *p* < 0.001. (**d**) Remaining amount of TAX in the mice cervicovaginal tract over time after administration of TAX, PAA-TAX and PAA-βCD/PAA-TAX nanogel. (**e**) Bioluminescence images of tumor tissue after different treatment. Adapted from [49]. Copyright© 2019 WILEY-VCH Verlag GmbH & Co. KGaA, Weinheim.

Compared with nanogels using conventional crosslinkers, drug-crosslinked nanogels showed unique advantages such as high drug loading content and improved drug release profile [81]. Zhu et al. designed a paclitaxel (TAX)-crosslinked nanogels for cervical cancer therapy [49]. They first synthesized βCD- and TAX-grafted poly(acrylic acid) (PAA), giving PAA-βCD and PAA-TAX, respectively. As βCD and TAX can form supramolecular interaction under hydrophobic condition, PAA-βCD and PAA-TAX may form nanogels (PAA-βCD/PAA-TAX) by virtue of such interaction (Figure 3a). DLS results indicated that the hydrodynamic size of the nanogel was 71.3 nm, and the size remained almost the same after storing for 60 days. As esterase can cleave the ester bonds, PAA-βCD/PAA-TAX nanogel may dissociate in the presence of esterase. The drug release profile indicated that under acidic condition with esterase, TAX had the highest release rate (Figure 3b). Flow cytometry study showed that PAA-βCD/PAA-TAX nanogel can be effectively internalized by HeLa cells. To evaluate the in vitro anti-cancer efficacy of nanogels, Hela, U14 cervical carcinoma cells expressing GFP (U14-GFP) and TAX-resistant HeLa (HeLa/TAX) cells were chosen as the model. The results demonstrated the satisfactory anti-cancer effect against all of these cells, indicating that nanogels can not only kill normal cancer cells, but also drug-resistant cancer cells (Figure 3c). The permeability of PAA-βCD/PAA-TAX nanogel into cells was then studied, and the results indicated that the nanogels can open the junction between epithelial cells. For cervical cancer treatment, drug retention in the cervicovaginal tract was critical for the therapeutic effect. An in vivo study showed that PAA-βCD/PAA-TAX nanogel had a significantly longer retention time than both TAX and PAA-TAX after vaginally administration (Figure 3d). In vivo therapeutic study indicated that the nanogels had highest tumor inhibition rate, and no obvious side effect was observed for such treatment (Figure 3e). Another example of drug-crosslinked nanogel was developed by Wang et al. [57]. They designed a Pt(IV) prodrug-crosslinked nanogel (TSCPNs) for cancer therapy. TSCPNs were prepared by polymerization of a Pt(IV) prodrug, followed by coating with amphiphilic copolymer TPGS1000. GSH can reduce TSCPNs and trigger the release of cisplatin. Such nanogels showed good anti-cancer efficacy both in vitro and in vivo. Zhu et al. also designed a Pt(IV)-crosslinked nanogels for chemotherapy [58]. Such nanogels were prepared via in situ polymerization of zwitterionic monomers and Pt(IV) crosslinker. A hypoxia-responsive drug tirapazamine (TPZ) was encapsulated into the nanogels. When the nanogels were internalized into tumor cells, GSH can degrade the nanogels and release Pt drugs and TPZ. As solid tumor had the feature of hypoxia, TPZ can further be reduced into cytotoxic TPZ radical to enhance the anti-cancer effect.

### 3.2. Gene Therapy

#### 3.2.1. Gene-Crosslinked Nanogels

**Figure 4:**
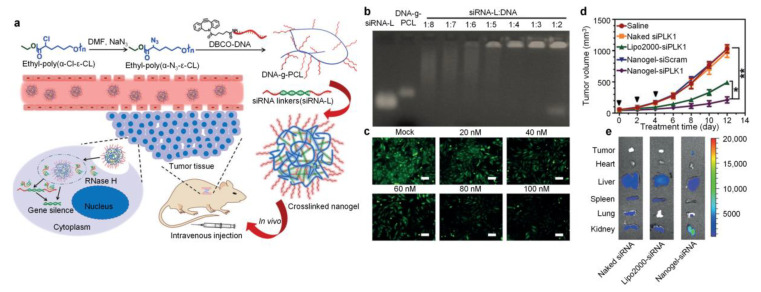
(**a**) Schematic illustration of siRNA-crosslinked nanogel formation and siRNA delivery process. (**b**) Characterization of nanogels with different siRNA-L to DNA ratio by 0.5% agarose gel electrophoresis. (**c**) Fluorescence microscopy images of U2OS·EGFP cells transfected with nanogels under different concentrations. The scale bars represent 100 μm. (**d**) Tumor volume change of MDA-MB-231 xenografted tumor-bearing mice over time after different treatment. * *p* < 0.05; ** *p* < 0.01. (**e**) Fluorescence images of major organs from tumor-bearing mice treated with different samples labeled with Cy5.5. Adapted from [51]. Copyright© 2018 WILEY-VCH Verlag GmbH & Co. KGaA, Weinheim.

Gene therapy is a promising tool to treat cancer, which inhibits the gene expression and changes the siRNA sequences [82]. However, the lack of an efficient carrier with low toxicity for gene delivery restricts the further application of gene therapy in clinic use. To address such issue, Zhang et al. designed a DNA-grafted poly(ε-caprolactone) (DNA-*g*-PCL) for siRNA delivery [51]. DNA-g-PCL can incorporate siRNA linkers (siRNA-L) to form crosslinked nanogel via electrostatic interactions. Such nanogels can effectively kill cancer cells after being internalized into cells (Figure 4a). The interaction between DNA-g-PCL and siRNA-L was studied via agarose gel electrophoresis. The results indicated that nanogels can be formed at the siRNA-L to DNA ratio ranging from 1:8 to 1:2 (Figure 4b). DLS results showed that the higher ratio of siRNA-L to DNA, the larger the size nanogels will have. Thus, the ratio of 1:6 was chosen as the candidate for further studies as the nanogel had satisfactory siRNA loading content and relatively small size. Such nanogels can be internalized into cells, which was confirmed by both confocal imaging and flow cytometry. In addition, an MTT assay revealed that the nanogels had good biocompatibility. The capability of gene silencing for nanogels was then studied by using enhanced green fluorescent protein (EGFP)-expressed U2OS (U2OS·EGFP) cells. With increasing the nanogel concentration, the fluorescence intensity of EGFP from U2OS·EGFP cells gradually decreased, indicating the successful EGFP silencing by nanogel (Figure 4c). Western blot analysis also showed that EGFP expression in U2OS cells was significantly suppressed, further demonstrating the gene editing capability of nanogels in cells. To study the efficiency of a nanogel-based siRNA delivery system for cancer therapy, DNA-g-PCL was incorporated with polo-like kinase 1 (PLK1) to give the corresponding nanogels. Compared with naked siPLK1 and Lipo2000-siPLK1, nanogel-siPLK1 showed the highest tumor inhibition rate, indicating its good in vivo anti-cancer effect (Figure 4d). In vivo biodistribution studies indicated that tumor accumulation of nanogel-siPLK1 was higher than that of naked siPLK1 and Lipo2000-siPLK1 (Figure 4e). These data demonstrated the superior anti-cancer efficiency of nanogel-siPLK1 over other treatments.

Another work reported by the same group used DNA-g-PCL to incorporate anti-Hsp70 siRNA (siHsp70), and the formed nanogel was coated with polydopamine (PDA) and PEG to give PEG-PDA-nanogel (PP-NG) [59]. Upon being internalized into cancer cells, PDA shell may be degraded and siHsp70 will be released to suppress the generation of Hsp70. Because of the down regulation of Hsp70, the photothermal therapy (PTT) induced by PP-NGs under laser irradiation may have better efficacy, leading to cell apoptosis under low temperature. Such low temperature PTT showed good therapeutic efficiency with low side effect.

#### 3.2.2. Environmental Responsive Nanogels for Gene Therapy

Although gene therapy has shown great promise in the field of cancer therapy, the off-target gene expression in normal tissue may also lead to severe side effects [83]. Therefore, development of environmentally responsive nanogel for tumor-specific gene delivery is in high demand to minimize the side effect of gene therapy. Sun et al. designed a disulfide bond containing nanogels which composed of thiolated dextrin and PEI [60]. PEI in the nanogels can load Bcl2 siRNA, which is an antiapoptosis factor in malignant cells. Such nanogels may be degraded in the presence of GSH, leading to the release of loaded siRNA. Agarose gel electrophoresis assay showed that siRNA may only be released under high concentration of GSH. Cellular studies indicated that nanogels had good biocompatibility and can be effectively internalized into cells. Both in vitro and in vivo therapeutic study demonstrated the good anti-cancer efficiency of siRNA-loaded nanogels.

**Figure 5:**
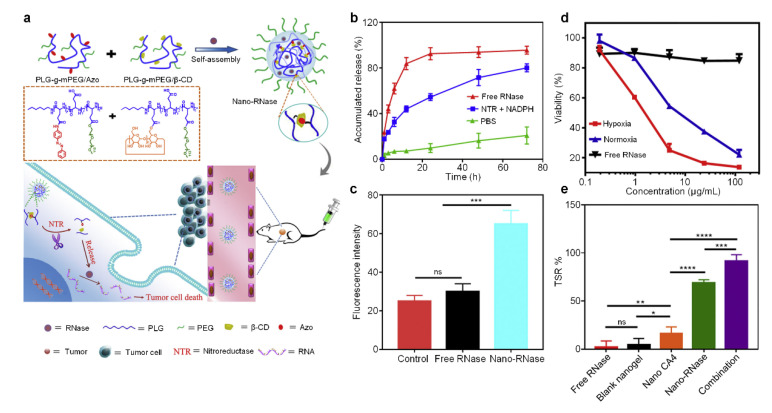
(**a**) Schematic illustration of preparation of Nano-RNase for hypoxia-responsive gene delivery. (**b**) In vitro RNase release profiles of nano-RNase under different condition. (**c**) Flow cytometry quantitative analysis of 4T1 cells incubated with free RNase or nano-RNase. (**d**) Cell viability of 4T1 cells incubated with different concentrations of Nano-RNase or free RNase under hypoxia or normoxia. (**e**) In vivo tumor suppression rate (TSR) of different treatments. * *p* < 0.05; ** *p* < 0.01; *** *p* < 0.001; **** *p* < 0.0001, ns represents no significant differences. Adapted from [61]. Copyright© 2020 Elsevier.

Hypoxia is one of the most commonly features in solid tumors, which has been widely utilized to develop activatable or smart DDS [84]. Based on such feature, Tang et al. designed a hypoxia-sensitive supramolecular nanogel for tumor-specific delivery of ribonuclease A (RNase) [61]. Such nanogels were composed of PEG-grafted poly(L-glutamic acid) modified with Azo (PLG-g-mPEG/Azo) and βCD (PLG-g-mPEG/βCD), respectively. The nanogels were formed via the supramolecular interaction between Azo and βCD, and RNase can be loaded into nanogels via electrostatic interaction. The hypoxic environment of a solid tumor may stimulate the expression of nitroreductase (NTR), which can cleave the Azo in the presence of NADPH. Thus, under hypoxic conditions, such a nanogel can be degraded and the loaded RNase will be released (Figure 5a). The ratio of PLG-g-mPEG/Azo to PLG-g-mPEG/βCD was set as 1:1, because under such ratio the nanogels had smallest hydrodynamic size. TEM images showed that the formed nanogels had a spherical morphology, and the nanogels would be degraded under a hypoxic condition. RNase was then loaded onto the nanogels to give Nano-RNase. When the ratio of PLG-g-mPEG/Azo to PLG-g-mPEG/βCD was set as 1:1, the nanogels had the highest RNase loading content and efficacy. DLS results indicated that Nano-RNase had a hydrodynamic size of 134 nm. The release profile of RNase was then studied. Under hypoxic conditions, 75% RNase was released after 72 h, while only 19.7% RNase can be released from Nano-RNase under normoxic condition (Figure 5b). Such a phenomenon can be explained by the degradation of Nano-RNase under hypoxic conditions. In addition, the activity of released RNase was completely remained compared with pure RNase. Cellular uptake of Nano-RNase was studied by confocal fluorescence imaging and flow cytometry. Flow cytometry analysis indicated that Nano-RNase can be effectively internalized into 4T1 cells, while only few free RNase can be internalized (Figure 5c). Confocal fluorescence imaging also confirmed the similar results. In vitro cytotoxicity showed that both PLG-g-mPEG/Azo and PLG-g-mPEG/βCD had good biocompatibility. Nano-RNase showed obvious cytotoxicity against 4T1 cells under both hypoxic and normoxic conditions, and the IC50 under hypoxia (1.7 μg/mL) was much lower than that under normoxia (7.6 μg/mL) (Figure 5d). In vivo anti-cancer efficacy of Nano-RNase was then studied. Pharmacokinetic profile studies showed that Nano-RNase had a prolonged blood circulation time compared to free RNase, which can be attributed to the long blood circulation of nanogels. For in vivo anti-cancer study, Combretastatin A4 (CA4) used as a vascular disrupting agent which can elevate hypoxia level in tumor site was co-delivered with RNase [85]. Compared with free RNase and Nano-CA4, Nano-RNase showed a higher tumor suppression rate (TSR) (68.7%), indicating its superior anti-cancer effect. In addition, combining with Nano-CA4, Nano-RNase can achieve even higher TSR (91.7%), which confirmed that higher hypoxia level may lead to better therapeutic efficiency (Figure 5e). H&E staining further demonstrated that abnormal cell nuclei and tumor tissue necrosis were clearly seen from combination therapy-treated tumor.

### 3.3. Enzyme Dynamic Therapy

Most therapeutic methods including chemotherapy and radiotherapy still face the issue of low selectivity and severe side effects [86]. In contrast, chemodynamic therapy (CDT) which utilizes hydrogen peroxide (H_2_O_2_) to generate cytotoxic ROS such as singlet oxygen (^1^O_2_) and the hydroxyl radical has gained increasing attention because of its high tumor specificity [87]. Until now, numerous nanomaterials such as MnO_2_ nanosheet and metal-organic frameworks (MOFs) have been developed for CDT [88,89].

**Figure 6:**
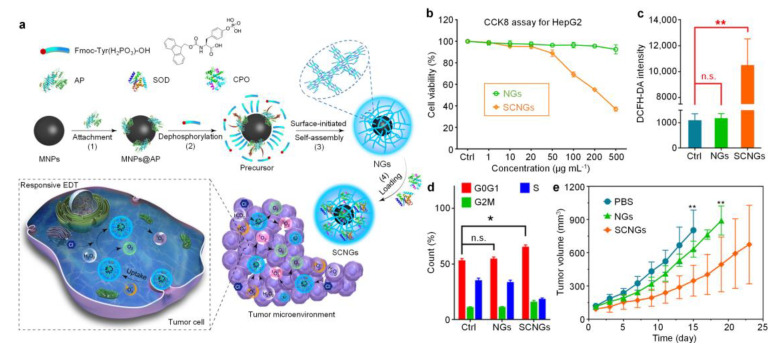
(**a**) Schematic illustration of SCNGs (superoxide dismutase (SOD) and chloroperoxidase (CPO) loaded onto nanogels (NGs)) preparation and mechanism of responsive enzyme dynamic therapy (EDT). (**b**) Cytotoxicity of NGs and SCNGs to HepG2 cells after 24 h incubation determined by cell counting kit-8 (CCK8) assay. (**c**) Flow cytometry analysis of fluorescence of reactive oxygen species (ROS) detector in HepG2 cells under different treatments. (**d**) Flow cytometry analysis of effects of SCNGs on cell cycles. HepG2 cells were treated by PI staining. The control, NGs and SCNGs groups represent HepG2 cells incubated with phosphate-buffered saline (PBS), NGs and SCNGs for 24 h, respectively. (**e**) Tumor volume change of hepatocellular carcinoma patient-derived xenograft (HCC PDX) mouse model under different treatments. The error bars represent standard deviation of six different measurements (*n* = 6). * *p* < 0.05; ** *p* < 0.01; n.s. represents no significant differences. Adapted from [53]. Copyright© 2019 Springer Nature.

Inspired by the concept of CDT, Wang et al. purposed the concept of enzyme dynamic therapy (EDT) which used enzyme to catalyze H_2_O_2_ for ^1^O_2_ generation [53]. They used tyrosine to coat monodispersed Fe_3_O_4_ nanoparticles (MNPs) to give shell crosslinked MNP nanogels (NGs). Then, superoxide dismutase (SOD) and chloroperoxidase (CPO) were loaded onto NGs to obtain SCNGs. After being internalized into cancer cells, SOD of SCNGs can catalyze ^·^O_2_^−^ into H_2_O_2_ in the presence of proton. H_2_O_2_ was further transferred into ^1^O_2_ under catalysis of CPO in the presence of chloride ion. The highly reactive ^1^O_2_ subsequently cause the death of cancer cells (Figure 6a). TEM and scanning electron microscopy (SEM) images showed that SCNGs had spherical morphology. SCNGs had a hydrodynamic size of 250 nm determined by DLS. Both Fourier transform infrared spectroscopy (FT-IR) measurements and thermogravimetry (TG) analysis indicated that the enzymes have been successfully loaded onto nanogels. ^1^O_2_ can be generated by SCNGs in the presence of H_2_O_2_ and NaCl, which was confirmed by the fluorescence change of the singlet oxygen sensor green (SOSG). Confocal fluorescence imaging also showed that SCNGs can generate ^1^O_2_ within HepG2 cells. For HepG2 cells, SCNGs showed obvious cytotoxicity, while NGs showed almost no cytotoxicity (Figure 6b). In contrast, both SCNGs and NGs showed good biocompatibility to normal hepatic HL-7702 cells. 5-chloromethyl-2′,7′-dichlorodihydrofluorescein diacetate acetyl ester (DCFH-DA) was then used to detect ROS generation within HepG2 cells. The results indicated that SCNGs-incubated cells had much higher fluorescence intensity than that of the control and NGs group, indicating higher ROS generation for SCNGs than NGs (Figure 6c).

The mechanism of SCNGs-induced cell apoptosis was then studied. They utilized γH2AX assay to study the DNA double-strand breaks, and the red fluorescence in the cells indicated that SCNGs-treated cells suffered the DNA damage. Flow cytometry analysis further demonstrated that SCNGs obstructed the cell cycle progression and interrupted the G0/G1 checkpoint transition, leading to the cell apoptosis (Figure 6d). In vivo anti-cancer study was then conducted by using a HepG2 tumor-bearing mouse model. The results showed that SCNGs had a much higher tumor inhibition rate than NGs. To further evaluate the anti-cancer efficacy of SCNGs, hepatocellular carcinoma (HCC) patient-derived xenograft (PDX) mouse model was used in the study. SCNGs showed satisfactory tumor inhibition rate, while NGs showed almost no inhibition to such a kind of tumor (Figure 6e). These data demonstrated the superior anti-cancer efficiency of SCNGs.

Based on such concept, the same group developed a neutrophil-mimic nanogels for enhanced EDT [50]. Such nanogels were also prepared by coating crosslinked tyrosine on the surface of MNP, followed by loading CPO. As MNPs had the feature of superparamagnetic, the nanogels can generate heat under an alternating magnetic field (AMF). When the nanogels were internalized into cancer cells, AMF may induce the nanogels to generate heat, which further elevate the ROS level of cancer cells and generate more H_2_O_2_. The CPO of nanogel can catalyze H_2_O_2_ into ^1^O_2_, thus leading to the death of cancer cells. Such work further demonstrated the feasibility of EDT, which expanded the therapeutic method for cancer.

## 4. Crosslinked Nanogels for Cancer Diagnosis and Imaging-Guided Cancer Therapy

Precise and personalized treatment can provide better therapeutic efficiency than traditional therapy, and are the trends in the field of cancer treatment [90]. To achieve such a goal, powerful diagnostic methods are in high demand as real-time monitoring of the therapeutic process is a fundamental requirement. Thus, development of imaging-guided cancer therapeutic systems has gained increasing attention in recent years [91]. By virtue of the advantages of crosslinked nanogels, they have been utilized for constructing variety of nanotheranostic systems. In this section, we summarized the applications of nanogels for cancer imaging and imaging-guided therapy. The general properties of nanogels discussed in this section were summarized in Table 3.

### 4.1. Cancer Imaging

Traditional diagnostic methods such as X-ray computed tomography (CT) imaging and magnetic resonance imaging (MRI) have been widely applied in clinics. However, the low signal-to-background ratio (SBR) of these imaging modalities limited their further application [92]. To improve such issues, various contrast agents have been developed to enhance the SBR [93]. Crosslinked nanogels have also been designed for the contrast agents of CT or MRI imaging.

Gold nanoparticles have been demonstrated as a good CT contrast agent. Shi et al. designed gold nanoparticles-based crosslinked nanogels for CT imaging [67]. Polyethyleneimine (PEI)-coated gold nanoparticles were first prepared via reducing HAuCl_4_ by NaBH_4_ in the presence of mPEG-modified PEI. After that, 1-ethyl-3-[3-(dimethylamino)propyl] carbodiimide hydrochloride (EDC) activated γ-polyglutamic acid (γ-PGA) was crosslinked by the gold nanoparticles to give γ-PGA-coated crosslinked gold nanoparticles (γ-PGA-[(Au^0^)_200_-PEI·NH_2_-mPEG] NGs). The X-ray attenuation intensity of such nanoparticles was much higher than that of Omnipaque, a commonly used CT contrast agent. The cellular uptake of nanogels was then studied, and the results indicated that γ-PGA-[(Au^0^)_200_-PEI·NH_2_-mPEG] NGs had a much higher cellular uptake than [(Au^0^)_200_-γ-PGA] NPs, which may be attributed to the softness and admirable fluidity of nanogels. In vivo CT imaging of tumor was then evaluated. γ-PGA-[(Au^0^)_200_-PEI·NH_2_-mPEG] NGs can effectively accumulate into tumors and light up the tumor site.

**Figure 7:**
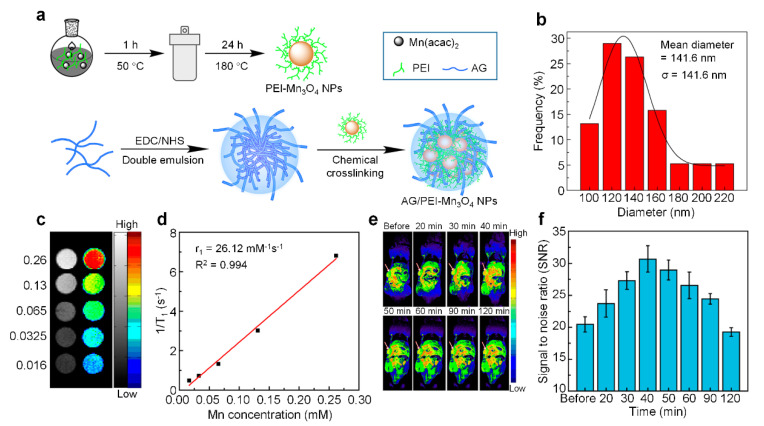
(**a**) Schematic illustration of preparation of AG/PEI-Mn_3_O_4_ NPs. (**b**) Size distribution histogram of AG/PEI-Mn_3_O_4_ NPs. (**c**) T_1_-weighted MRI images of AG/PEI-Mn_3_O_4_ NPs under different concentrations. (**d**) Linear fitting of 1/T_1_ with Mn concentration of AG/PEI-Mn_3_O_4_ NPs. (**e**) In vivo MRI images of U87MG xenografted tumor bearing mice at different time points post-injection of AG/PEI-Mn_3_O_4_ NPs. (**f**) Signal-to-noise ratio (SNR) of tumor area at different time points calculated from MRI images in (**e**). Adapted from [68]. Copyright© 2018 American Chemical Society.

Apart from CT imaging, MRI is also a conventional imaging modality. Similar with CT imaging, MRI has the issue of low sensitivity as well [94]. Therefore, development of a high-performance contrast agent is needed. The same group designed a kind of similar nanogels for in vivo MRI [68]. PEI-coated Mn_3_O_4_ nanoparticles (PEI-Mn_3_O_4_ NPs) were first prepared by reducing Mn(acac)_2_ with PEI. Then, EDC/N-hydroxysuccinimide (NHS)-activated alginate (AG) was crosslinked by mixing with PEI-Mn_3_O_4_ NPs to give the desired nanogels (AG/PEI-Mn_3_O_4_ NGs) (Figure 7a). Such nanogels had a spherical morphology with hydrodynamic size of 141.6 nm (Figure 7b). The nanogels showed good stability in aqueous solution. The 1/T_1_ value showed a linear correlation with Mn concentration, indicating the feasibility of quantification study (Figure 7c). In addition, the r1 value of AG/PEI-Mn_3_O_4_ NGs (26.12 mM^−1^s^−1^) was much higher than that of PEI-Mn_3_O_4_ nanoparticles (1.34 mM^−1^s^−1^), which can be attributed to the larger dimension of AG/PEI-Mn_3_O_4_ NGs than PEI-Mn_3_O_4_ nanoparticles (Figure 7d). AG/PEI-Mn_3_O_4_ NGs showed good biocompatibility, and much higher MR contrast enhancement than PEI-Mn_3_O_4_ nanoparticles in living U87MG cells. AG/PEI-Mn_3_O_4_ NGs were then applied for in vivo tumor imaging. After intravenous (i.v.) injection of AG/PEI-Mn_3_O_4_ NGs into tumor-bearing mice, the MR signal in the tumor site gradually increased and reached a maximum at t = 40 min post-injection (Figure 7e). At such a time point, the tumor SBR can reach 30.7, much higher than that before injection (20.4) (Figure 7f). In contrast, the MR signal in the tumor area of PEI-Mn_3_O_4_ nanoparticles-injected mice showed no significant difference before and after injection. These results showed that AG/PEI-Mn_3_O_4_ NGs can effectively accumulate into tumor site after i.v. injection, and successfully enhance the SBR of tumor.

Multimodal imaging which combines two or more imaging modalities into one system usually has better imaging effect than any single imaging modality [95]. To combine the advantages of above two nanogel systems, Shi et al. designed a CT/MRI dual-modal imaging nanogel system [69]. They conjugated DTPA-Gd onto the PEI-coated gold nanoparticles, and then used such nanoparticles to crosslink AG to give the final nanogels, AG/PEI-Au-Gd NGs. As gold nanoparticles and DTPA-Gd were good CT and MRI contrast agents, such nanogels can be used for CT/MRI dual-modal imaging of tumors in living mice.

As an emerging imaging modality, optical imaging has gained increasing attention owing to its high safety and sensitivity, capability of multichannel imaging [96]. Sanyal et al. designed a kind of clickable nanogel for in vitro fluorescence imaging [62]. The nanogels were prepared by crosslinking a thermo-responsive copolymer. Such copolymer contained maleimide groups on the side chains, which can react with thiol-containing compounds. The copolymers may form aggregates under high temperature, and they can be subsequently crosslinked by 2,2′-(ethylenedioxy)diethanethiol to form nanogels. The nanogels can then react with thiol-containing fluorophores and tumor targeting groups to endow nanogels with the capability of fluorescence cell imaging and tumor targeting.

**Figure 8:**
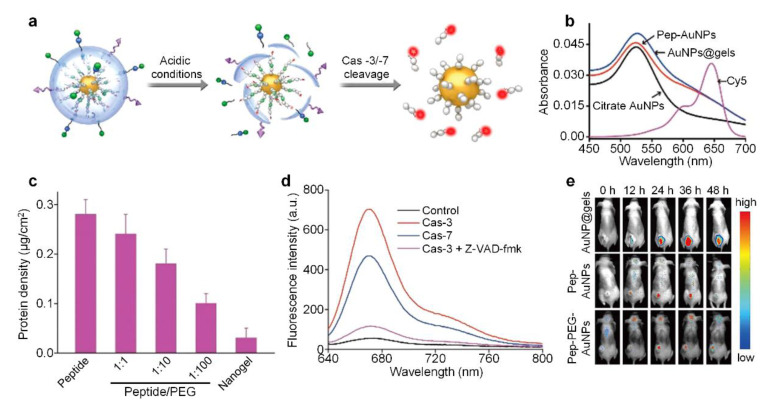
(**a**) Schematic illustration of caspase activity detection by AuNP@gel probes. (**b**) Absorption spectra of citrate-coated AuNPs, pep-AuNPs, AuNPs@gels and Cy5 solutions. (**c**) Quantitative analysis of total absorbed proteins on the surface of AuNPs by bicinchoninic assay (BCA). (**d**) Fluorescence spectra of AuNPs@gels in the presence of Caspase-3 and -7 under different pH. (**e**) Fluorescence images of tumor bearing mice under different treatments. Adapted from [70]. Copyright© 2019 American Chemical Society.

Compared with “always-on” probes, the environmental responsive probes which emit signal under specific stimulus showed great potential in the field of tumor imaging [97]. Liu et al. designed a kind of crosslinked nanogel for in vivo imaging caspase activity [70]. To synthesize the nanogels, gold nanoparticles were first modified with vinyl group and Cy5-linked peptides. Then, the surface of nanoparticles was crosslinked by crosslinkers with double bonds via radical polymerization to form the nanogels. At the same time, zwitterionic and amine groups, and folic acid were modified onto the surface to give the final nanogel, AuNP@gel. The shell of such nanogels may be degraded under acidic condition, and caspase-3 or -7 can further cleave the peptide to release the Cy5. When linked onto the surface of gold nanoparticles, the fluorescence of Cy5 was quenched. Such a process can thus turn on the fluorescence of Cy5, which can be used as the indicator of caspase-3 or -7 (Figure 8a). AuNPs@gels had a board absorption ranging from 450 to 700 nm, which can be attributed to the absorption of gold nanoparticles and Cy5 (Figure 8b). Atomic force microscopy (AFM) results indicated that the nanogels had a spherical morphology. The stealth effect of AuNPs@gels was then studied by polyacrylamide gel electrophoresis (PAGE) and bicinchoninic assay (BCA). The PAGE results indicated that AuNPs@gels had the lowest protein absorption amount compared with peptide- or PEG-modified AuNPs. The BCA data also showed similar results, AuNPs@gels had the lowest protein density on the surface (Figure 8c). The responsiveness of AuNPs@gels to caspases were then studied. Under physiological conditions, only weak fluorescence can be detected. Such fluorescence can be activated only in the presence of caspase-3 or -7, and the detection of limit (LOD) for caspase-3 and -7 were calculated as 0.23 and 0.65 nM, respectively (Figure 8d). AuNPs@gels were then applied for fluorescence cell imaging by using DOX-treated cells. As DOX can induce the expression of caspase-3 and -7, AuNPs@gels showed strong fluorescence signal in such cells. In vivo fluorescence of a tumor by AuNPs@gels was then conducted. AuNPs@gels, pep-AuNPs or pep/PEG-AuNPs was first injected into mice via the tail vein, followed by the injection of DOX. The fluorescence images of mice were then captured. The results indicated that AuNPs@gels-injected mice had the highest fluorescence signal in the tumor site, demonstrating the in vivo activation of AuNPs@gel (Figure 8e). These results confirmed the caspase-sensing capability of AuNPs@gels.

### 4.2. Imaging-Guided Cancer Therapy

Using imaging technique to real-time monitor the therapeutic efficacy and guide the therapeutic process is crucial in the development of theranostic systems. By virtue of the advantages of optical imaging, phototheranostics have gained tremendous attention in recent years [98]. Zhao et al. designed a nanogel based on Cu_2−x_S nanoparticles [71]. Chlorin e6 (Ce6)-conjugated PEI (PEI-Ce6) was coated onto the surface of Cu_2−x_S nanoparticles, followed by crosslinking PEI by 3-(carboxypropyl)triphenylphosphonium bromide (TPP-COOH) to form the nanogels (CCeT NPs). Cu_2−x_S nanoparticles and Ce6 had good photothermal and photodynamic efficiency, respectively, endowing nanogels with good phototherapy effect. In addition, CCeT NPs had satisfactory fluorescence intensity. These features make CCeT NPs good candidates for fluorescence imaging guided phototherapy. Another phototheranostic system based on Ce6 was developed by Wu et al. [63]. They used Ce6 to crosslink polyhedral oligomeric silsesquioxane (POSS) via amidation reaction. The formed nanogels were then conjugated with PEG on the surface to give POSS-PEG-Ce6 NPs. Because the nanogels were crosslinked by covalent bonds, they had excellent stability under different conditions. POSS-PEG-Ce6 NPs can be effectively internalized into cells and showed strong fluorescence signal within cells. Under laser irradiation, POSS-PEG-Ce6 NPs-incubated HeLa cells showed obvious cytotoxicity, demonstrating their good in vitro PDT effect. In vivo studies indicated that POSS-PEG-Ce6 NPs can effectively accumulate into tumor of mice, and showed good PDT efficacy against tumor. Lu et al. designed a microwave-responsive nanogel for on-demand drug delivery and enhanced cancer therapy [64]. Such nanogels were synthesized by crosslinking zwitterionic monomer (2-((2-(methacryloyloxy)ethyl) dimethylammonio)acetyl) (phenylsulfonyl) amide (MEDAPA) with ethyleneglycol dimethacrylate (EGDMA) and bis(acryloyl)cystamine (BAC). After that, transferrin and Cy5 were then conjugated onto the surface of nanogels, and DOX can be loaded into the nanogel. Under high temperature, the hydrodynamic size of nanogels can increase from 168.5 nm to 212.4 nm. Such a process significantly increased the release rate of DOX, thus enhancing the therapeutic efficacy. In vivo fluorescence imaging indicated that nanogels can effectively accumulate into tumor site. The therapeutic study results showed that DOX-loaded nanogels with the help of microwaves had best therapeutic effect compared with other treatments such as nanogels without microwave and free DOX.

**Figure 9:**
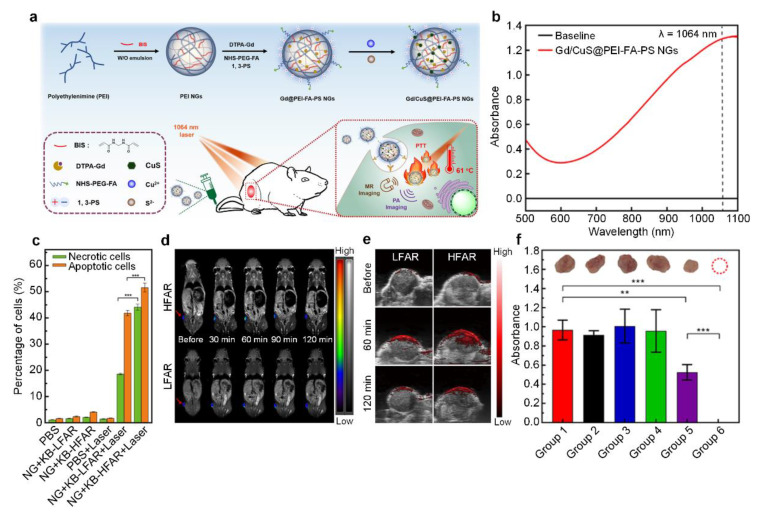
(**a**) Schematic illustration of Gd/CuS@PEI-FA-PS NGs for magnetic resonance imaging (MRI) and photoacoustic (PA) imaging-guided PTT. (**b**) Absorption spectrum of Gd/CuS@PEI-FA-PS NGs. (**c**) Flow cytometry analysis of necrotic and apoptotic KB cells under different treatments. (**d**) In vivo T1-weighted MR (**d**) and PA (**e**) images of high-level FA receptor (FAR) expression (HFAR) and low-level FAR expression (LFAR) tumor-bearing mice in different post-injection time points. (**f**) Tumor weights from mice under different treatments for 24 days. ** *p* < 0.01; *** *p* < 0.001. Adapted from [72]. Copyright© 2020 American Chemical Society.

Multimodal imaging-guided phototherapy system can also be developed by nanogels for more precise diagnosis than single imaging modality-based theranostics. Shi et al. designed a nanogel for MRI and photoacoustic (PA) dual-modal imaging-guided PTT [72]. Such nanogels were synthesized by Michael addition reaction between branched PEI and N,N′-methylenebis(acrylamide) (BIS). Then, the as-prepared DTPA-Gd, NHS-PEG-FA and 1,3-propanesultone were conjugated onto the nanogels. After that, Cu ion was loaded onto the nanogels and reacted with S^2-^ to give CuS-loaded nanogels (Gd/CuS@PEI-FA-PS NGs) (Figure 9a). TEM images showed that nanogels had a spherical morphology. Energy-dispersive spectroscopy (EDS) results indicated that the nanogels contained Cu, Gd and S elements, demonstrating the successful preparation of Gd/CuS@PEI-FA-PS NGs. Absorption spectrum of Gd/CuS@PEI-FA-PS NGs was determined by ultraviolet–visible (UV–vis) spectroscopy, and a board absorption band ranging from 600 to 1100 nm was detected. The high absorption coefficient at 1064 nm endowed it with good candidate for near infrared (NIR-II) phototherapy (Figure 9b). The T_1_ MR and PA signal of Gd/CuS@PEI-FA-PS NGs were then measured. Both signals showed a linear relationship between the concentration of nanogels, demonstrating their ability of quantitative analysis. In addition, the r_1_ value of Gd/CuS@PEI-FA-PS NGs (11.66 mM^−1^s^−1^) was much higher than that of Magnevist (4.56 mM^−1^s^−1^), a commercial MRI contrast agent. Gd/CuS@PEI-FA-PS NGs had good photothermal effect, which their temperature can reach 69.2 °C under 1064 nm laser irradiation. Gd/CuS@PEI-FA-PS NGs showed good biocompatibility under dark condition, and had high cytotoxicity towards KB cells under laser irradiation. For both high-level FA receptor (FAR) expression (HFAR) and low-level FAR expression (LFAR) KB cells, flow cytometry analysis indicated that large number of necrotic and apoptotic cells were observed under Gd/CuS@PEI-FA-PS NGs incubation with laser irradiation (Figure 9c), further demonstrating its good in vitro anti-cancer efficacy.

Gd/CuS@PEI-FA-PS NGs were then applied for in vivo MRI and PA imaging of tumor. After i.v. injection of Gd/CuS@PEI-FA-PS NGs, both MRI and PA signals in the tumor area of HFAR and LFAR tumor-bearing mice increased gradually, indicating the accumulation of nanogels into the tumor (Figure 9d,e). The highest signal for both MRI and PA imaging in the tumor of mice was observed at t = 60 min post-injection. In addition, the signals from HFAR tumor-bearing mice were significantly higher than that from LFAR tumor bearing mice, demonstrating the FA-mediated specific targeting of Gd/CuS@PEI-FA-PS NGs. Gd/CuS@PEI-FA-PS NGs were then utilized for in vivo PTT. For the phosphate-buffered saline (PBS) and nanogel without laser groups, the tumor grew rapidly. In contrast, obvious inhibition for tumor growth was observed in nanogels with laser groups, and the HFAR group showed the highest tumor inhibition rate among all the groups (Figure 9f). These data indicated that Gd/CuS@PEI-FA-PS NGs can effectively light up tumor and inhibit tumor growth by PTT.

Among all the therapeutic methods, chemotherapy is still the major choice in clinical applications. Shi et al. designed a DOX-loaded nanogels based on branched PEI and BIS mentioned above [73]. Fe_3_O_4_ nanoparticles were coated onto the surface of nanogels to endow the nanogels with capability of MRI. Then, DOX was loaded into the nanogels to achieve the final Fe_3_O_4_/PEI-Ac NGs/DOX. Such drug-loaded nanogels showed good T_1_-weighted MR signal, which their r_1_ value was 2-fold higher than ultrasmall Fe_3_O_4_ nanoparticles. DOX can be gradually released from nanogels, and the release profile showed a pH-dependent manner, which its release rate under pH 5.5 was faster than that under pH 7.4. In vitro and in vivo studies indicated that Fe_3_O_4_/PEI-Ac NGs/DOX had good anti-cancer efficacy, and can delineate tumor tissue by its MRI signal.

**Figure 10:**
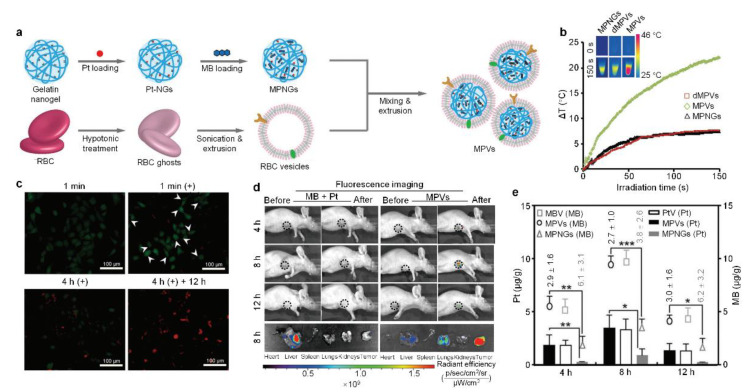
(**a**) Schematic illustration of preparation of red blood cell membrane-coated nanogels. (**b**) photothermal effect of pre-irradiated membrane-coated nanogels (dMPVs), MPVs and MPNGs under laser irradiation. (**c**) Fluorescence images of 4T1 cells incubated with MPVs for different times with or without laser irradiation. (**d**) Representative fluorescence images of MPVs- and free drugs-injected mice before and after laser irradiation at different time points. (**e**) Intratumoral concentrations of MB and Pt from tumor bearing mice treated with MBVs, PtVs, MPNGs, or MPVs for 4, 8, and 12 h. * *p* < 0.05; ** *p* < 0.01; *** *p* < 0.001. Adapted from [65]. Copyright© 2018 WILEY-VCH Verlag GmbH & Co. KGaA, Weinheim.

Cell membrane-camouflaged nanoparticles have been widely studied in the field of cancer theranostics. The coated cell membrane can prolong the circulation time and enhance tumor accumulation of nanoparticles [99,100]. Li et al. designed a red blood cell (RBC) membrane-coated nanogels (MPVs) for cancer fluorescence imaging and chemotherapy [65]. To prepare MPVs, gelatin nanogels were first synthesized, Pt and methylene blue (MB) were then subsequently loaded onto the nanogels to give MPNGs. RBC membrane extracted from RBCs was coated onto the surface of MPNGs to give MPVs (Figure 10a). MPVs had a spherical morphology with the size of ~150 nm. SDS-PAGE analysis indicated that membrane proteins from RBCs such as CD47 was well reserved on the surface of MPVs. After laser irradiation, the membrane permeability of MPVs was increased, leading to faster drug release rate compared with MPVs without pre-irradiation. MPVs showed a satisfactory photothermal effect, while the pre-irradiated MPVs (dMPVs) and MPNGs showed a low photothermal effect because of the release of MB (Figure 10b). In vitro cell imaging showed that with laser irradiation, MPVs can effectively release MB within cells after incubation with cell for only 1 min., while no such release can be detected without laser irradiation. LIVE/DEAD viability assay was then used to detect the membrane disintegration of cells. After incubating MPVs with 4T1 cells for 1 min, almost no cytotoxicity was observed. However, obvious disintegration of membranes was detected after laser irradiation, indicating the release of drugs. After 4 h incubation, increased cell death was observed, and almost no live cell was detected after additional 12 h incubation (Figure 10c).

After demonstrating the good anti-cancer efficacy in vitro, MPVs were applied for in vivo cancer phototheranostics. After i.v. injection of MPVs for 8 h, the PA signal in tumor area was significantly enhanced compared with before the injection. While, no such enhancement was observed for free MB and Pt-injected mice. The fluorescence images of MPVs-injected mice were captured before and after laser irradiation at different post-injection time points. For MPVs-injected mice, the fluorescence signal in the tumor site was increased after laser irradiation at all the time points. Such a phenomenon can be attributed to the release of MB from MPVs after laser irradiation, leading to the recovery of fluorescence signal. In contrast, no such fluorescence increase was observed for free MB and Pt-injected mice (Figure 10d). The fluorescence image of major organs resected from mice at t = 8 h post-injection also indicated that the fluorescence signal of tumor for laser-irradiated mice was much higher than that of non-irradiated mice. In addition, the drug concentration in the tumor site for MPVs-injected mice was 2–3 fold higher than free drug and MPNGs-injected mice (Figure 10e). Because of the high drug delivery efficiency, MPVs showed best therapeutic effect among all the treatments. H&E staining results indicated that no damage to normal organs was observed for MPVs-treated mice, demonstrating the safety of such treatment.

Activatable drug delivery system can release drug under specific environment, which can elevate the therapeutic efficacy and reduce side effect [101]. Meng et al. designed a pH/GSH dual-stimuli-responsive nanogels for MRI-guided chemotherapy [74]. Fe_3_O_4_ nanoparticles were served as the core of nanogels, while the shell was synthesized by crosslinking hexachlorocyclotriphosphazene (HCCP) with curcumin (CUR) and bis-(4-hydroxyphenyl)-disulfide (HPS). Ce6 was then loaded onto the surface of nanogels. Under acidic conditions, CUR can be cleaved from the shell, leading to the dissociation of shell and release of CUR and Ce6. Such process can also be triggered by GSH, which can cleave the disulfide bonds of HPS. Such a design endowed nanogels with tumor-specific drug-delivery capability.

## 5. Conclusions and Outlook

In recent years, crosslinked nanogels have shown great promise in the field of cancer imaging and therapy. Compared with traditional micelles and liposomes, nanogels have a covalently crosslinked nanostructure, endowing nanogels with better physiological stability, multifunctionality and improved drug-release profile. The nanogels usually have a hydrodynamic size of tens to hundreds of nanometers, making them effectively accumulate in tumor sites. In addition, nanogels show good biocompatibility both in vitro and in vivo, indicating their potential in clinical translation. The nanogels can be used for anti-cancer drug and gene delivery. Such drug or gene-loaded nanogels have an improved release profile. In addition, novel therapeutic methods such as EDT can be designed based on the structure of nanogels, providing good therapeutic efficacy with minimized side effect. In addition to drugs, contrast agents can be conjugated or loaded onto nanogels. Such nanogels have been widely applied for imaging-guided cancer therapy. Nanogels loaded with photosensitizers can be used for phototheranostics, while Fe_3_O_4_-coated or Gd-conjugated nanogels are good candidates for MRI. Owing to the multifunctionality of nanogels, activatable chemotherapy-based nanotheranostic systems may also be developed.

In the field of cancer imaging, numerous advantages have been shown from these crosslinked nanogels. Crosslinked nanogels have better stability than micelles and liposomes, which can prevent burst release during circulation, thus improving the tumor accumulation and drug-delivery efficiency. Some nanogels were reported to had higher accumulation in a tumor site compared with other major organs including liver and spleen [60,74], which was seldom reported for micelles and liposomes [102]. In addition, fluorophores can be easily conjugated onto the surface of nanogels by virtue of their multifunctionality. Such a design was superior to encapsulation of fluorophores within nanoparticles as their fluorescence signal may be quenched due to aggregation [103]. Gd complexes are usually conjugated onto the nanogels for T_1_-weighted MRI. Compared with Gd-conjugated micelles, nanogels may retain their MRI signal better in the circulation owing to their higher physiological stability [72].

In the aspect of cancer therapy, higher tumor accumulation leads to higher drug-delivery efficiency and lower toxicity towards normal tissues. Compared with other surface engineered nanomaterials such as silica nanoparticles [104], nanogels may have better biodegradability, and are easier to develop on-demand DDS. These features make loaded drugs be easily released from nanogels. On the other hand, compared with other biodegradable nanomaterials such as polyester nanoparticles, nanogels usually have higher stability [105]. Thus, the flexibility of structures makes nanogels not only have good physiological stability, but also can release drugs in the desired site.

Although crosslinked nanogels have shown great potential in the field of cancer theranostics, several issues need to be resolved to further push forward the clinical translation of nanogels. As nanogels have a crosslinked structure, they usually have poor biodegradability compared with micelles and liposomes. Thus, the metabolic period may be much longer than micelles or liposomes. Such a process may cause long-term toxicity which hinders the clinical translation of nanogels. To improve such an issue, nanogels constructed by biodegradable materials or crosslinkers can be a rational choice to prepare biodegradable nanogels. Therefore, preparing nanogels with such a feature can be one of the research focuses in future study. Another critical issue for clinical translation is the productivity and reproducibility of nanogel preparation. However, to the best of our knowledge, there is no study on how to increase the productivity of nanogels at an industrial scale, and all the preparation methods are limited on the laboratory scale. In addition, no study on the reproducibility of nanogel preparation is reported yet. Thus, studies on these two issues are also in high demanded.

## Figures and Tables

**Table 1:** Summary of the preparation of nanogels by types, inorganic cores, materials, crosslinkers and crosslinking interactions.

Type	Inorganic Core	Materials	Crosslinker	Crosslinking Interaction	References
Organic		Alginate	Ca ion	Electrostatic interaction	[54]
		Dendritic blocks	TMP-SH	Covalent bond	[55]
		Hyaluronic acid	Keratin	Hydrogen bond	[56]
		PAA-βCD	PAA-TAX	Supramolecular interaction	[49]
		-	Pt(IV) prodrug	Covalent bond	[57]
		-	PPM	Covalent bond	[58]
		DNA-*g*-PCL	siRNA	Electrostatic interaction	[51]
		DNA-*g*-PCL	siRNA	Electrostatic interaction	[59]
		Dextrin-SH	PEI-SH	Covalent bond	[60]
		PLG-g-mPEG/βCD	PLG-g-mPEG/Azo	Supramolecular interaction	[61]
		poly(PEGMEMA-co-MaMA)	2,2′-(ethylenedioxy)diethanethiol	Covalent bond	[62]
		POSS	Ce6	Covalent bond	[63]
		MEDAPA	EGDMA/BAC	Covalent bond	[64]
		Gelatin	glutaraldehyde	Covalent bond	[65]
Inorganic	MNP-NH_2_	Alginate	-	Covalent bond	[66]
	MNPs	Tyrosine	-	π-π interaction	[53]
	MNPs	Tyrosine	-	π-π interaction	[50]
	AuNPs	γ-PGA	PEI	Covalent bond	[67]
	Mn_3_O_4_ NPs	Alginate	PEI	Covalent bond	[68]
	AuNPs	Alginate	PEI	Covalent bond	[69]
	AuNPs	Cy5/vinyl-labeled peptide	Glycerol dimethacrylate	Covalent bond	[70]
	Cu_2−x_S NPs	PEI	Ce6	Covalent bond	[71]
	CuS NPs	PEI	BIS	Covalent bond	[72]
	Fe_3_O_4_ NPs	PEI	BIS	Covalent bond	[73]
	Fe_3_O_4_ NPs	HCCP	CUR/HPS	Covalent bond	[74]

TMP-SH: trimethylolpropane tris(3-mercaptopropionate); TAX: paclitaxel; PPM: Pt(IV)-based prodrug monomer; PEI: polyethyleneimine; POSS: polyhedral oligomeric silsesquioxane; Ce6: Chlorin e6; MEDAPA: (2-((2-(methacryloyloxy)ethyl) dimethylammonio)acetyl) (phenylsulfonyl) amide; EGDMA: ethyleneglycol dimethacrylate; BAC: bis(acryloyl)cystamine; MNP: magnetic nanoparticles; AuNP: gold nanoparticles; PGA: polyglutamic acid; BIS: N,N′-methylenebis(acrylamide); HCCP: hexachlorocyclotriphosphazene; CUR: curcumin; HPS: bis(4-hydroxyphenyl)-disulfide.

**Table 2:** Summary of the nanogels for cancer therapy by loaded drug, loading capacity, responsiveness and animal study.

Type	Loaded Drug	Loading Capacity	Responsiveness	Animal Study	References
Chemotherapy	DOX/GL	1.2% (DOX)	pH	Yes	[54]
	DOX	5.7%	-	No	[55]
	DOX	54.1%	pH/GSH/trypsin	Yes	[56]
	DOX	18.2%	pH/GSH	Yes	[66]
	TAX	20–30%	pH/esterase	Yes	[49]
	Pt(IV)	60.8%	GSH/ascorbic acid	Yes	[57]
	Pt(IV)/TPZ	8.06% (Pt)/9.12% (TPZ)	GSH	Yes	[58]
Gene therapy	siRNA	-	RNase H	Yes	[51]
	siRNA	-	pH/RNase H	Yes	[59]
	siBcl2	-	DTT	Yes	[60]
	RNase	23.5%	NTR	Yes	[61]
Enzyme dynamic therapy	-	-	·O_2_^−^/H_2_O_2_	Yes	[53]
	-	-	H_2_O_2_	Yes	[50]

DOX: doxorubicin; GL: glycyrrhizin; DTT: dithiothreitol; NTR: nitroreductase.

**Table 3:** Summary of the nanogels for cancer imaging and imaging-guided therapy by imaging modality, therapeutic method, responsiveness and animal study.

Type	Imaging Modality	Therapeutic Method	Responsiveness	Animal Study	References
Imaging	CT	-	-	Yes	[67]
	T_1_-MRI	-	-	Yes	[68]
	T_1_-MRI/CT	-	-	Yes	[69]
	FL	-	-	No	[62]
	FL	-	pH/caspases	Yes	[70]
Imaging-guided therapy	FL	PDT/PTT	-	Yes	[71]
	FL	PDT	-	Yes	[63]
	FL	Chemotherapy	Temperature	Yes	[64]
	T_1_-MRI/PA	PTT	-	Yes	[72]
	T_1_-MRI	Chemotherapy	pH	Yes	[73]
	PA/FL/PT	PDT/chemotherapy	Laser	Yes	[65]
	Fluorescence/T_2_-MRI	PDT/chemotherapy	pH/GSH	Yes	[74]

CT: X-ray computed tomography; MRI: magnetic resonance imaging; FL: fluorescence; PDT: photodynamic therapy; PTT: photothermal therapy; PA: photoacoustic.
